# Yarkovsky and YORP effects simulation on 3200 Phaethon

**DOI:** 10.1098/rsta.2024.0205

**Published:** 2025-02-27

**Authors:** Hiroki Senshu, Hirotomo Noda, Fumi Yoshida, Takashi Ito, Maximilian Hamm, Sean Marshall

**Affiliations:** ^1^ Planetary Exploration Research Center, Chiba Institute of Technology, 2-17-1 Tsudanuma, Narashino, Chiba 275-0016, Japan; ^2^ RISE Project, National Astronomical Observatory of Japan, Oshu, Iwate 023-0861, Japan; ^3^ The Graduate University for Advanced Studies, SOKENDAI, Hayama, Kanagawa 240-0193, Japan; ^4^ Department of Basic Sciences, University of Occupational and Environmental Health Japan, 1-1 Iseigaoka, Yahata, Kitakyusyu, Fukuok 807-8555, Japan; ^5^ Center for Computational Astrophysics, National Astronomical Observatory of Japan, 2-21-1 Osawa, Mitaka, Tokyo 181-8588, Japan; ^6^ College of Science and Engineering, Chubu University, 1200 Matsumoto-cho, Kasugai, Aichi 487-8501, Japan; ^7^ Planetology and Remote Sensing, Institute of Geological Sciences, Freie Universität Berlin, Malteserstr. 74-100, Berlin 12249, Germany; ^8^ Institute of Planetary Research, German Aerospace Center (DLR), Rutherfordstr. 2, Berlin 12489, Germany; ^9^ Florida Space Institute, University of Central Florida, 12354 Research Pkwy, Orlando, FL 32826-0650, USA

**Keywords:** Phaethon, Yarkovsky, YORP, thermal modelling, numerical simulation

## Abstract

We carry out a numerical study on the thermal behaviour of asteroid 3200 Phaethon, the parent body of the Geminid meteor shower. Phaethon’s orbit is highly eccentric, with the perihelion distance as small as 0.14 au. During the perihelion passage the surface temperature rises so much that the reaction force of the thermal radiation from its surface affects Phaethon’s orbital and spin motion. We evaluate this thermal effect precisely by numerically modelling the thermal variation during an orbital period. We find that the thermal effect, both on the orbital motion and spin, changes its sign near the perihelion owing to the geometric configuration of the spin axis direction relative to the Phaethon–Sun direction. Consequently, the canonical expression of the thermally induced momentum is not applicable for the case of bodies on a highly eccentric orbit such as Phaethon. Nevertheless, the resulting orbital-averaged thermal effect is consistent with ground-based observation and previous estimates.

This article is part of the theme issue ‘Major advances in planetary sciences thanks to stellar occultations’.

## 1. Introduction

Found in 1983, 3200 Phaethon is a potentially hazardous asteroid. Its size is estimated to be 5−6 km [1–3]. Currently, Phaethon has a large inclination (22 degrees) and eccentricity (0.89), which brings its perihelion to 0.14 au and thus closer to the Sun than the orbit of Mercury. Phaethon exhibits cometary activity near its perihelion [4–7] and is dynamically associated with the Geminid meteor shower [8]. The coming Japanese flyby mission DESTINY+ is scheduled to observe Phaethon at a close range within this decade [9,10].

Phaethon’s shape, spin rate and spin pole direction are required to construct a thermal model, and this information has been estimated from ground-based photometric and radar observations (e.g. [1–3,11,12]). Understanding thermal conditions on the asteroid surface are highly relevant since (i) the thermal radiation is an observable value and is thus a diagnostic value in size estimation [13,14]; (ii) if the thermal inertia is obtained, the size of grains covering the asteroid can be estimated [15–18]; and (iii) the temperature distribution can affect the motion and spin of the asteroid [19,20].

Owing to thermal inertia, the hottest part on the surface of a small body is located at local afternoon. For prograde spinning asteroids, this part lies on the trailing hemisphere, and for retrograde spinning asteroids on the leading hemisphere. The reaction force from thermal photon emission pushes the small body. While this force is small, thermally induced momentum can accumulate over longer timescales and change the orbital motion, for prograde spinning asteroids in the direction of the orbit spiralling away from the sun, for retrograde spinning, asteroids spiralling inwards. This effect is known as the Yarkovsky effect [19,20]. Also, if the small body has an asymmetric shape, the anisotropic recoil force of thermal radiation from the surface will cause a net torque on it, an effect known as the Yarkovsky–O’Keefe–Radzievskii–Paddack (YORP) effect [19,20], which causes a long-term change to the rotation period and axis orientation. To understand the current and past dynamical state of asteroids, a thermophysical model of the surface temperature variations is required.

Phaethon, specifically, is known to have a large eccentricity and to come closer to the Sun than that of Mercury’s orbit. Thus, temperature distribution and thermal photon emissions change significantly within orbital motion [2,3]. In addition, if the dynamical state of Phaethon changes from thermal photon emission, it would affect the prediction of the Geminid meteor shower occurrence.

Therefore, in this study, we revisit the thermal model of Phaethon with a precise model of its shape and dynamics and investigate the resulting Yarkovsky and YORP effects on its spin state and orbit. We provide an overview of our numerical model for simulating the seasonal and diurnal temperature variations and on how to estimate the thermal force from the resulting thermal state. The numerical results are presented in §3. A discussion and summary of our results are given in §§4 and 5, respectively.

## 2. Model description

### (a) Numerical model

We simulate the surface thermal evolution of Phaethon by using a shape model consisting of triangular polygons. In this model, we solve the one-dimensional thermal diffusion equation for each polygon:

(2.1) $$\rho C_{p}\frac{\partial T}{\partial t}=\frac{\partial }{\partial z}\left(k\frac{\partial T}{\partial z}\right)+F_{s},$$

where T is the temperature, ρ is the density, t is the time, z is the depth from the surface, $C_{p}$ is the specific heat, k is the thermal conductivity and $F_{s}$ is the surface energy flux given as

(2.2) $$F_{s}=\left\{ \begin{array}{ll} \xi \frac{\left(1-A\right)S_{⊙}}{{\left(r/1\text{au}\right)}^{2}}\cos\theta -\varepsilon \sigma T_{s}+\sum_{i}F_{i}\varepsilon \sigma T_{s,i}^{4},\;\; & z=0,\\ \;\;\;\;\;\;\;\;\;\;\;\;\;\;\;\;\;\;\;\;\;\;\;\;\;\;\;\;\;\;\;\;\;\;\;\;\;\;\;\;\;\;\;\;\;\;\;\;\;\;\;\;\;\;\;\;\;\;\;\;\;\;\;\;\;\;\;\;\;\;\;\;\;\;\;\;\;\;\;\;\;0,\;\; & z\neq 0, \end{array}\right.$$

where A is the bond albedo, $S_{⊙}$ is the solar energy flux at 1 au, r is the solar distance, θ is the solar incident angle, ε is the surface emissivity and σ is the Stephan–Boltzmann constant. The casting of shadows on the surface is accounted for by setting ξ=0 if the solar radiation is cut by another polygon, and ξ=1 otherwise. The thickness of the top layer is set to be 1/50th that of the thermal skin depth given as $\sqrt{\tau k/\rho C_{p}}$, where τ denotes the spin period for diurnal temperature change, and the thickness of each successive layer increases by a factor of 1.01. A portion of the thermal flux from a polygon might be absorbed by another polygon. This self-heating effect [21–25] is accounted for by the third term on the right-hand side of equation (2.2); $F_{i}$ is the view factor which represents the fraction of energy leaving one facet and reaches the other [21–25].

Senshu *et al*. [25] showed that small-scale roughness changes the apparent surface temperature distribution. However, in this study, we ignore sub-polygon scale roughness to avoid introducing too many free parameters. This might result in an underestimation of the thermal torque caused by the tangential YORP effect [26,27], but it is known that the Yarkovsky effect is not affected by small-scale roughness [25,28].

In previous thermophysical models of asteroids, it was a common way to fix asteroid's position in the inertial coordinate system, and the diurnal thermal evolution was simulated repeatedly to obtain an equilibrium surface temperature distribution [29–31]. However, MacLennan *et al*. ([32], see also [28]) reported that the surface temperature distribution estimated from a fixed-position model can differ from that estimated from a model that factors in orbital motion to consider the seasonal effect. This discrepancy is caused by a seasonal wave in the subsurface temperature distribution, which may be exacerbated by Phaethon’s large eccentricity and its tilting spin pole. Thus, in this study, we follow orbital revolutions of Phaethon iteratively, taking into account both the diurnal and the seasonal effects to obtain an equilibrium subsurface temperature distribution. We then use the result as an initial condition to precisely simulate the thermal evolution for one orbital revolution (June 2021–November 2022).

### (b) Yarkovsky and YORP effects

The thermally induced momentum flux (Yarkovsky effect) acting on the surface is given by $p=\varepsilon \sigma T_{s}^{4}/2\pi c$$\text{[J}/{\text{m}}^{2}\cdot sr]$, where *c* is the speed of light. However, for a rough surface, a portion of the photons emitted from an area will be absorbed by another area, thus negating its reaction force. To take the reabsorption effect into account, we first render a thermal image, after which we integrate each ‘pixel value’ to obtain the total photon energy flux directed toward the observer. The self-heating effect is naturally taken into account in this method [25].

The YORP effect is estimated in a similar manner to that of the thermally induced momentum (Yarkovsky effect). The thermally induced torque (YORP effect) **t** is estimated by $t=-\left(r\times d\right)\;\varepsilon \sigma T^{4}$, where  r is the position vector of the area from the small body’s barycentre and d is the unit vector of the radiation direction. As is done for the Yarkovsky effect, we integrate the torque vector after rendering thermal images with various observation directions to obtain the thermal YORP effect on the small body.

#### (i) Gauss’s planetary equations

The change in orbit owing to a small perturbation is estimated by solving Gauss’s planetary equations [33]. These equations describe the relationship between the time derivative of orbital elements and external perturbation forces; not using disturbing potential as the ordinary Lagrange’s planetary equations do. We calculate the time derivative of orbital elements at each time step by using the set of thermally unperturbed orbital elements and numerically derived thermal forces.

The total change of orbital elements is derived from the time integration of the derivatives. This method is not applicable for long-term integration because Gauss’s planetary equations are a nonlinear system, but the result of the Yarkovsky effect on the orbital motion is so small on the considered time scale that we treat it as a perturbation in this study [33].

If an asteroid’s shape is spherical and its spin pole is orthogonal to the orbital plane, the hottest area on the surface remains in the orbital plane, resulting in the Yarkovsky effect affecting only the along-track orbital motion. However, if the spin pole tilts, the hottest area moves out of the orbital plane direction. Solving Gauss’s planetary equations allows us to evaluate the effect of the Yarkovsky effect on the direction out of the orbital plane.

### (c) Phaethon’s parameters

Table 1 lists the parameters used in this study. The shape model used was determined from 12 days of radar observation from two apparitions (2007 and 2017), approximately 200 light curves (149 nights; 18 apparitions from 1989 to 2022) and eight occultation observations from three apparitions (Marshall *et al.,* in preparation). The volume estimated from the shape model is $\left(6.7\pm 0.8\right)\times 10^{10}\;{\text{m}}^{3}$. The mass of Phaethon is calculated to be $1.12\times 10^{14}\;\text{kg}$ for the bulk density of $1670\;\text{kg}/{\text{m}}^{3}$ [3]. The density of Phaethon was estimated in several studies but they are consistent within errors. For example, [34] proposed 1580 ± 450 kg/m^3^. Note that the mass is scaled by the density, meaning that the estimate of the Yarkovsky and YORP effects can be changed depending on the density. The moment of inertia is estimated to be $3.69\times 10^{20}\;\text{kg}\cdot {\text{m}}^{2}$ from the shape model and the uniform density. Note that the moment of inertia is also scaled to the assumed density.

**Table 1.** Thermo-physical parameters of Phaethon.

parameter	value	note
volume	(6.7 ± 0.8) × 10^10^ m^3^	calculated from the shape model
density	1670 ± 470 kg/m^3^ 1580 ± 450 kg/m^3^	Hanuš *et al.* [3] MacLennan *et al.* [32]
mass	1.12 × 10^14^ kg	calculated from the volume and the density
moment of inertia	3.69 × 10^20^ kg m^2^	calculated from the shape model and the density
albedo	0.048	bond albedo
emissivity	0.90	canonical value
thermal inertia	600 J/Ks^1/2^ m^2^	characteristic value
production of density and heat capacity	10^6^ J/K m^3^	ρCp

The moment of inertia of Phaethon is calculated from its mass, shape and the assumption of uniform interior density. The bond albedo (0.048) is taken from [2,3] and the value of the emissivity in thermal infrared wavelength is taken to be the canonical value (0.90).

The characteristic thermal inertia of Phaethon is set to $600\;\text{J}/\text{K}{\text{s}}^{1/2}\;{\text{m}}^{2}$ [2,3,34] in this study, but we vary the thermal inertia as a free parameter.

Note that the surface density calculated from the thermal inertia does not need to be the same as the bulk density. The surface could be covered by regolith and/or the surface layer would be highly modified by solar radiation and/or space weathering. For 162173 Ryugu, the target body of Hayabusa2, the remotely sensed surface properties were not the same as those measured with the returned sample in a laboratory [16]. This is because the surface skin layer of the Ryugu sample was fragile owing to space weathering and, consequently, was crushed in the sampling capsule when it entered into Earth’s atmosphere [35].

The ephemeris of Phaethon was obtained from the JPL Horizons system (https://ssd.jpl.nasa.gov/horizons/). Our ephemeris was obtained on 17 January 2024 and its version is JPL#783.

## 3. Numerical results

**Figure 1.**
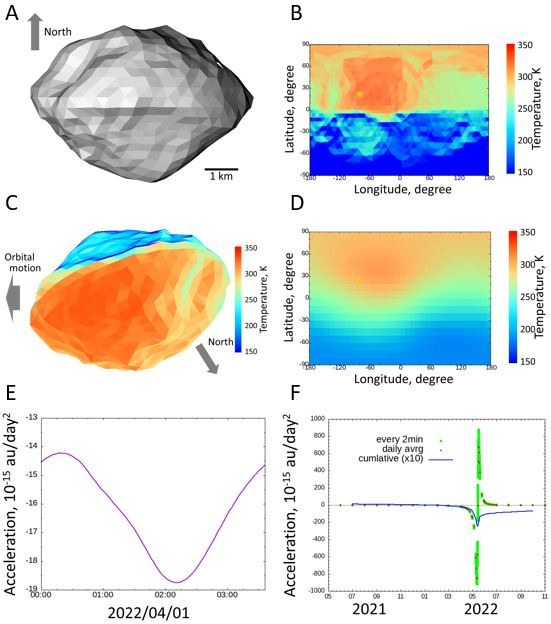
Numerical procedure to estimate the Yarkovsky effect working on 3200 Phaethon. (A) Shape model of Phaethon used in this study. (B) Map of surface temperature. (C) Thermal image made from the panels (A) and (B). (D) Map of the brightness temperature. (E) Time series of the thermally induced momentum in one spin rotation. (F) Time series of the Yarkovsky effect for one orbital revolution of Phaethon. Orange circle on (B) and (D) represents the sub-solar point. See the text for more detail.

Figure 1A–F show a typical result of the thermal model to demonstrate the steps of the data analysis process. The thermal inertia is assumed to be 600 J/Ks^1/2^ m^2^ in this case.

Figure 1A shows the shape of Phaethon observed from a distant point along the direction of (lon, lat) = (−90, 0), the top and bottom corresponding to north and south, respectively. Phaethon has an equatorial bulge, and its northern and southern hemispheres have a conical shape, a so-called double-top shape; some depression can be seen. Figure 1B shows the surface temperature distribution recorded at 02:30:00 UTC, 1 April 2022, in a longitude–latitude map. At this moment, Phaethon was 1.09au away from the Sun, and the sub-solar point was (lon, lat) = (−81.15, 25.45). This figure shows an obvious north–south temperature dichotomy. This is due to Phaethon’s double-top shape, where the solar incident angle is lowest in the northern hemisphere and higher in the southern hemisphere. The hottest area spreads towards the afternoon hemisphere owing to the thermal inertia effect. Figure 1C shows the surface temperature distribution observed from the Sun’s direction at the same time as figure 1B. The top is the direction of Phaethon’s orbital angular momentum, and the left-hand side is the leading hemisphere. Because Phaethon’s spin pole points downwards in this figure, the left- and right-hand sides correspond to the afternoon and morning hemispheres, respectively. This figure also shows an obvious north–south dichotomy in the temperature distribution, as explained above. The thermally induced momentum flux (Yarkovsky effect) towards the observer is given as the fourth-power mean of each pixel value in figure 1C. Figure 1D shows the brightness temperature as a function of the observation direction. This was obtained by integrating the surface temperature distribution, as shown in figure 1C, for various observation directions at the same time with figure 1C. The integration of figure 1C gives one of the points with figure 1D. It should also be noted that figure 1D resembles the surface temperature distribution (figure 1B), but they are not the same. The direction of maximum thermally induced momentum flux is obtained in the direction (lon, lat) = (−40, 40), which is shifted from the sub-solar point (lon, lat) = (−81.15, 25.45). This offset causes the along-track thermal acceleration/deceleration (the Yarkovsky effect). The offset is caused by not only the thermal inertia effect but also the uneven shape. The difference between the surface temperature distribution and the Yarkovsky effect would be obvious for a very uneven body, such as Itokawa, Eros and 67 P/Churyumov–Gerasimenko.

The omnidirectional integration of figure 1D gives the Yarkovsky effect acting on Phaethon at that moment. We estimated the time-series Yarkovsky effect for one spin rotation, which is shown in figure 1E. We can see that the Yarkovsky effect is not constant during one spin rotation. This comes from the temperature distribution heterogeneity owing to Phaethon not having a simple spherical shape.

The time-series acceleration/deceleration was obtained by carrying out the procedure described above for each orbit revolution. We first calculated the Yarkovsky effect during one spin rotation on the 1st of each month, then more frequently when Phaethon was close to its perihelion (figure 1F). As is shown in figure 1F, the Yarkovsky effect was not constant during one orbital revolution. The same procedure was carried out for various thermal inertias to check the parameter dependency.

**Figure 2.**
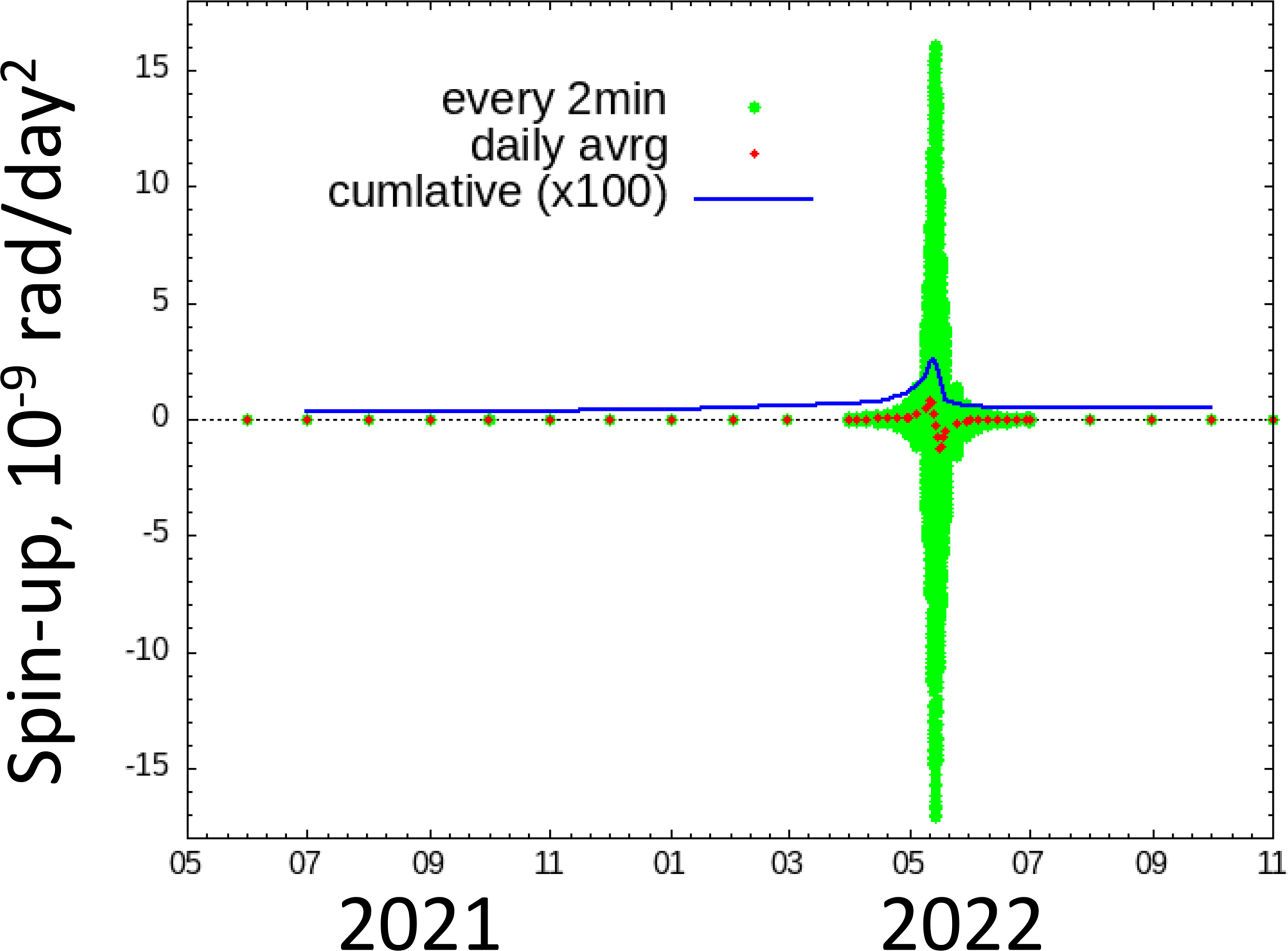
The thermal torque (YORP effect) working on Phaethon as a function of time for one orbital period of Phaethon.

Figure 2 shows the change in the rate of spin rotation caused by the thermal torque (YORP effect). The spin rotation change, ${\overset{˙}{\omega }}_{\text{spin}}$, is calculated from the spin pole direction component of the thermal torque $t_{\text{spin}}$ divided by the moment of inertia of the spin pole, I, i.e. ${\overset{˙}{\omega }}_{\text{spin}}=t_{\text{spin}}/I$. The time-series thermal torque is estimated using a similar procedure as that used for the Yarkovsky effect.

**Figure 3.**
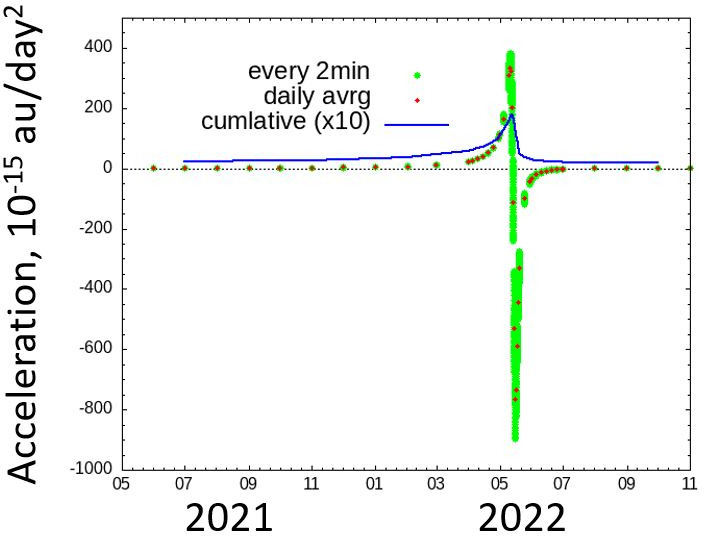
The thermally induced momentum towards out-of-plane direction as a function of time for one orbital period of Phaethon.

Figure 3 shows the out-of-plane component of the thermally induced momentum. The cumulative average value of the out-of-plane thermally induced momentum remains non-zero after one orbital revolution. This does not mean the Sun lay outside Phaethon’s orbital plane, but rather the shape or direction of orbital eclipse would change with the Sun at one of the focal points.

The resulting new position of Phaethon after one orbital period is shifted by −597 m along the orbit, −342 m radially and −128 m in a direction out of the orbital plane. Because the position shift is much smaller than the orbital error (similar to that of Phaethon’s size) it is undetectable from single astrometric observation. However, Phaethon’s orbital speed at aphelion becomes as low as 6.4 km/s and thus the ground-based occultation observation would be able to detect approximately a one-tenth second delay of the occultation event.

## 4. Discussion

Phaethon spins retrogradely, so the Yarkovsky effect tends to induce acceleration in the opposite direction to that of the direction of orbital motion. The Yarkovsky effect becomes stronger near the perihelion because the surface temperature becomes higher. For Phaethon, the along-track thermal force changes its sign near the perihelion (figure 1F). This is not because of the perihelion passage but because of the geometric configuration of the spin pole direction relative to the Phaethon–Sun direction. The sub-solar point on Phaethon moves across the equator from north to south just before the perihelion. At this moment, the northern hemisphere is on the leading hemisphere, while the southern hemisphere is on the trailing hemisphere. Thus, the hottest point on Phaethon moves from the leading hemisphere to the trailing hemisphere, resulting in strong deceleration before the perihelion, and acceleration after the perihelion. This effect is also enhanced by Phaethon’s shape. The north pole of Phaethon is flat, while the south pole is rather elongated, resulting in a ’brilliant-cut’ shape. This causes the deceleration before the perihelion passage to be suppressed compared to the case with a spherical shape.

**Figure 4.**
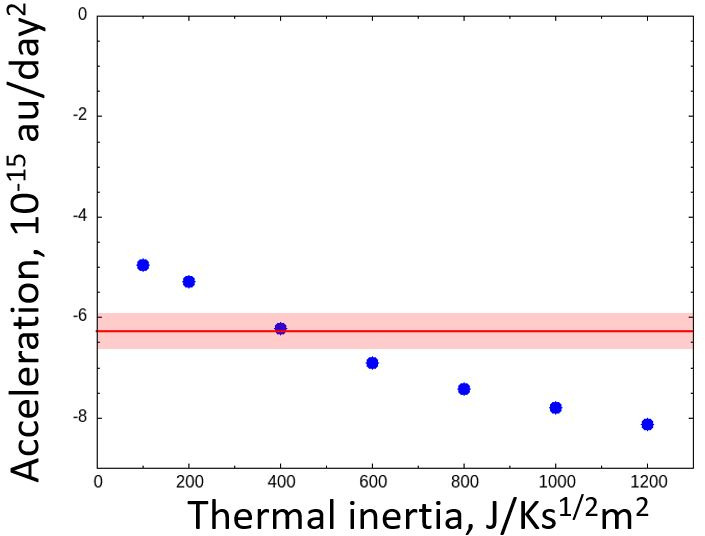
Along-track direction thermally induced momentum, averaged over one orbit of Phaethon as a function of thermal inertia. The hatched area represents the uncertainty (0.36 × 10–15 au/day^2^).

The orbit-averaged along-track deceleration given by our numerical simulation was similar to the value determined by the ground-based observation, denoted as $A_{2}$, which was $-6.29\times 10^{-15}\;\text{au}/{\text{day}}^{2}$ (JPL Horizons data). The $A_{2}$ value is usually used to express non-gravitational effects as a function of the solar distance using ${\left(A_{2}/\left(r/\text{1 au}\right)\right)}^{-d}$, where d is a constant. However, as shown in figure 1F, the along-track force changes its sign depending on the spin pole direction. Thus, the power-law formula of the along-track deceleration cannot be used to express the deceleration in this case. Figure 4 shows the orbit-averaged along-track deceleration as a function of the thermal inertia. Our numerical results are $-5.3\times 10^{-15}$, $-6.2\times 10^{-15}$ and $-6.9\times 10^{-15}\;\text{au}/{\text{day}}^{2}$ for the thermal inertias 200, 400 and $600\;\text{J}/\text{K}{\text{s}}^{1/2}{\text{m}}^{2}$, respectively. Our numerical result is consistent with the *$A_{2}$* value provided by NASA Horizons if the thermal inertia is between 200 and $600\;\text{J}/\text{K}{\text{s}}^{1/2}{\text{m}}^{2}$ or larger. It is known that the local shape of the asteroid can change the efficiency of Yarkovsky effect by several tens of per cent [36]. Thus, the consistency of the deceleration derived from our numerical study with the ground observations is corroborative evidence that Phaethon’s position shift along its orbit is caused by the reaction force of thermal radiation (the Yarkovsky effect), and no sporadic cometary activity is needed to explain the deceleration.

Marshall compiled ground-based observations dating from 1989 and found that the spin rate of Phaethon changes secularly with a rate of $\overset{˙}{\omega }=\left(3.7\pm 0.7\right)\times 10^{-8}\;\text{rad}/{\text{day}}^{2}$ [37]. The spin state of comets is known to change in response to their activity, but the spin rate change of Phaethon is so constant that it is hard to explain by sporadic and random activities. On the other hand, the YORP effect causes constant spin rate change over time scales longer than one orbital period.

The YORP effect depends on the shape of the asteroid. Specifically, the YORP effect caused by small-scale morphology is known as the tangential (T) YORP to distinguish it from from the normal (N) YORP caused by a simple surface [26,27]; T YORP can be one order of magnitude larger than N YORP. Thus, the one-order-of-magnitude discrepancy between our numerical results and ground observations is still reasonable. This gap can be filled by the precise *in situ* disc-resolved observations of the upcoming DESTINY+ mission [9,10].

MacLennan *et al*. [32] recently proposed that Phaethon has heterogeneity in its thermal inertia to explain the difference in the thermal flux from Phaethon’s surface for different epochs. If the thermos-physical properties change with locations, then this effect might be occurring owing to its sun-grazing orbit and/or its shape. A sun-facing hemisphere could be irradiated from the sun preferentially during its perihelion passage, causing space weathering, micro impacting and/or thermal cracking. The resulting regolith would be transported on the surface afterwards. Then, Yarkovsky and YORP effects can be affected. The heterogeneity of the surface is out of the scope of this study because it would introduce many free parameters; however, the effect is to be considered in the future studies.

The position shift of Phaethon owing to the Yarkovsky effect after one orbital period is of the order of several hundred metres. On the other hand, the occultation band shift for the event on 21 October 2022 at Hokkaido in Japan was shifted from the predicted occultation band southward by approximately the radius of Phaethon [38]. The distance of the position shift cannot be explained by the Yarkovsky effect only. This might suggest cometary activity during the last perihelion passage, or simply that there is an error in the prediction of the occultation band location.

Gauss’s planetary equations also give the change of Phaethon’s semi-major axis, $\overset{˙}{a}$. This value is important for determining the fate of small bodies and whether there is a possibility for them to harm the Earth system. Direct observation of $\dot{a}$ from the ground is difficult, but the value for 101955 Bennu has been determined to be −284.6 m/yr from the OSIRIS-REx mission observations [39]. The $\dot{a}$ value of 162173 Ryugu was not determined by the spacecraft’s observations but was estimated from numerical simulations to be −139 m/yr [40]. Ryugu is approximately twice as large as Bennu, and the difference in the $\dot{a}$ value between them is explained by this size difference.

**Figure 5.**
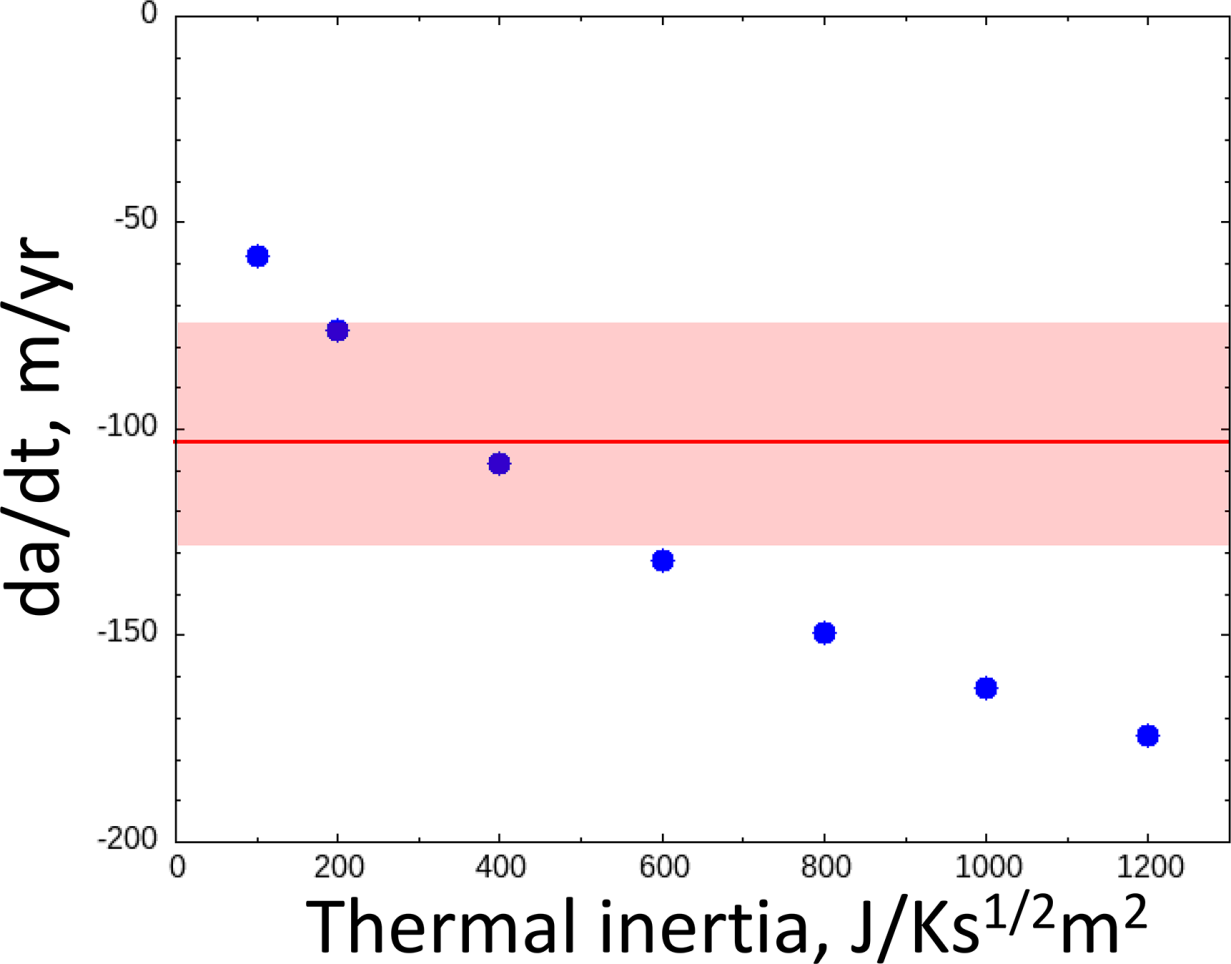
Change of the semi-major axis averaged over one orbit as a function of thermal inertia. The hatched area represents the uncertainty (28 m/yr).

Therefore, the $\overset{˙}{a}$ value of Phaethon, of which the diameter is approximately 6 km, is expected to be much smaller than that of these asteroids. However, its value was estimated in a previous study [9] to be $\overset{˙}{a}=-\left(6.9\pm 1.9\right)\times 10^{-4}\;\text{au}/\text{Myr}=-103\pm 28\;\text{m}/\text{yr}$. The $\dot{a}$ value from our model is not constant during one rotation. Figure 5 shows the orbit-averaged $\overset{˙}{a}$ value as a function of thermal inertia. The averaged value is similar to that in the previous study [3]. The difference of Phaethon’s $\overset{˙}{a}$ value from the expected size dependency should be a consequence of Phaethon’s large eccentricity and double-top shape. Thus, the effect of the perihelion passage should be taken into account when considering the fate of a small body and its potential to harm the Earth system.

## 5. Summary

We performed numerical simulations of the thermal evolution of Phaethon to determine the thermal effect on its motion. Our thermophysical model succeeded in explaining the along-track deceleration of Phaethon owing to the thermally induced momentum (the Yarkovsky effect). The spin-up of Phaethon was suggested, but the numerically estimated value was one order of magnitude weaker than the observed value. This is probably because of the T YORP effect caused by small-scale roughness on the surface which we cannot account for in our model. Thus, we expect that the disc-resolved imaging of the Japanese Phaethon mission DESTINY+ [9,10] will fill this knowledge gap.

Our numerical results also suggest that the tilted spin axis causes a reaction force of thermal radiation out of the orbital plane direction. However, the shift in the occultation band cannot be explained by the tangential Yarkovsky effect.

## Data Availability

The data were partly produced by using the SPICE toolkit developed by NASA JPL NAIF team [41].
